# Chromium Picolinate Regulates Bone Metabolism and Prevents Bone Loss in Diabetic Rats

**DOI:** 10.3390/molecules29050924

**Published:** 2024-02-20

**Authors:** Hongxing Zheng, Wenrui Yan, Mengli Shao, Shanshan Qi

**Affiliations:** 1 School of Biological Science and Engineering, Shaanxi University of Technology, Hanzhong 723000, China; zhenghongxing@snut.edu.cn (H.Z.); yanwenrui0105@163.com (W.Y.); shaomengli9722@163.com (M.S.); 2 State Key Laboratory of Qinba Biological Resources and Ecological Environment, Hanzhong 723000, China; 3 Shaanxi Black Organic Food Engineering Technology Research Center, Hanzhong 723000, China; 4 Qinba Mountain Area Collaborative Innovation Center of Bioresources Comprehensive Development, Hanzhong 723000, China; 5 Shaanxi Provincial Key Laboratory of Resource Biology, Hanzhong 723001, China; 6 Shaanxi Migukang Biotechnology Company, Xi’an 710018, China

**Keywords:** chromium picolinate, diabetic osteoporosis, bone metabolism, bone microstructure, bone transformation

## Abstract

Diabetic osteoporosis (DOP) is an abnormal metabolic disease caused by long-term hyperglycemia. In this study, a model rat of streptozotocin (STZ)-induced diabetes was established, and chromium picolinate (5 mg·kg^−1^) was given; the changes in blood glucose and body weight were detected before and after administration; and bone mineral density (BMD), bone morphology, bone turnover markers, inflammatory cytokines, and oxidative stress indicators were observed in each group. We found that after chromium picolinate (CP) intervention for 8 weeks, the blood glucose level was decreased; the BMD, the bone histomorphology parameters, and the pathological structure were improved; the expression of bone resorption-related proteins was downregulated; and the expression of bone formation-related proteins was upregulated. Meanwhile, serum antioxidant activity was increased, and inflammatory cytokine levels were decreased. In conclusion, CP could alleviate DOP by anti-oxidation, inhibition of bone turnover, anti-inflammation, and regulation of the OPG/RANKL/RANK signaling pathway. Therefore, CP has important application values for further development as a functional food or active medicine in DOP treatment.

## 1. Introduction

Diabetes is one of the world’s three major chronic noncommunicable diseases. Compared with healthy people, diabetic patients have a higher chance of suffering from cardiovascular diseases, and aging is accelerated; therefore, diabetes can induce many diseases and seriously affect health [1,2]. It is estimated that by 2045, there will be 700 million people living with diabetes worldwide [3]. Diabetic osteoporosis (DOP) is a secondary osteoporosis caused by hyperglycemia [4]. Patients with DOP have a low bone turnover rate, low bone mineral density (BMD), and low bone strength [5]. A survey of patients with diabetes showed that more than 35% of patients had symptoms of bone loss, and about 20% of them met the diagnostic criteria of osteoporosis, and in China, more than one-third of diabetic patients were suffering from osteoporosis [6].

Osteoporosis caused by diabetes mellitus is a systemic endocrine disease, and its pathogenesis is closely related to chronic inflammation and oxidative stress caused by long-term hyperglycemia [7]. Chronic inflammation and excessive ROS can promote osteoclast differentiation and formation, thus promoting bone absorption and inducing bone fractures [8,9,10]. Moreover, the OPG/RANK/RANKL signaling pathway can also regulate osteoclast differentiation, thus affecting bone remodeling and bone formation [11,12].

Chromium (Cr), as one of the essential trace elements in the human body, is related to the activity of glucose tolerance factor (GTF); has a positive effect on glucose intolerance and insulin resistance [13]; and participates in the metabolism of glucose, lipids, proteins, and nucleic acids [14,15]. Chromium picolinate (CP) is a kind of organic chromium with good stability. CP has the effects of lowering blood glucose, alleviating oxidative stress, lowering blood lipids, improving cardiovascular diseases, and promoting weight loss [16,17,18,19,20]. As it has good hypoglycemic effects, it is clinically used for diabetes treatment.

In our previous study, CP was found to improve kidney tissue damage induced by streptozotocin (STZ) in diabetic rats [21]. Based on its good hypoglycemic effect, as well as its anti-oxidant, anti-inflammatory, and anti-diabetic nephropathy effects, we speculated that it might have a protective effect against osteoporosis caused by diabetes. Therefore, we established a diabetic rat model and then administered CP to investigate the effect of CP on DOP. We hope this research can provide a new method for the treatment of diabetes-induced bone loss and osteoporosis.

## 2. Results

### 2.1. Chromium Picolinate Relieved Diabetic Symptoms in Diabetic Rats

**Figure 1:**
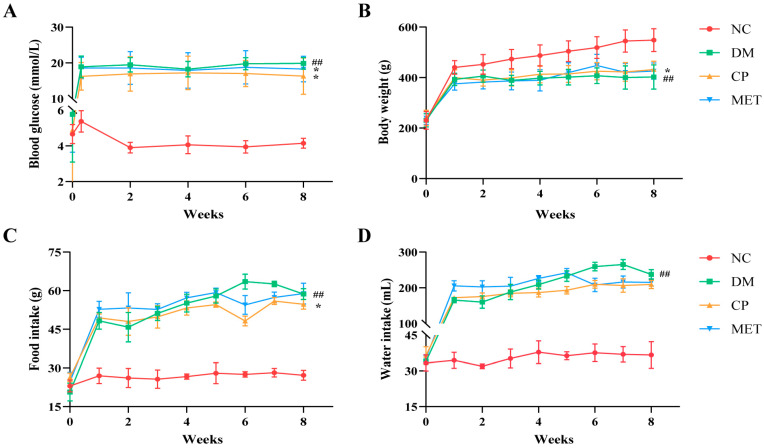
Blood glucose, body weight, food intake, and water intake of the rats in each group. (**A**) Blood glucose. (**B**) Body weight. (**C**) Food intake. (**D**) Water intake. NC, normal control group; DM, diabetic model group; CP, diabetic rats treated with CP (5 mg∙kg^−1^∙d^−1^); MET, diabetic rats treated with metformin (200 mg∙kg^−1^∙d^−1^). The values are presented as mean ± SD. In comparison to the NC group, (##) *p* < 0.01. In comparison to the DM group, (*) *p* < 0.05.

As shown in Figure 1, fasting blood glucose, food intake, and water intake in the DM group were markedly elevated (*p* < 0.01 vs. NC), and body weight was decreased (*p* < 0.01 vs. NC). After CP or MET administration, the fasting blood glucose levels in the CP and MET groups were obviously decreased (*p* < 0.05 vs. DM), and body weight was obviously elevated (*p* < 0.05 vs. DM). This suggests that CP could relieve the symptoms of diabetes.

### 2.2. Chromium Picolinate Increased BMD and Bone Strength

**Figure 2:**
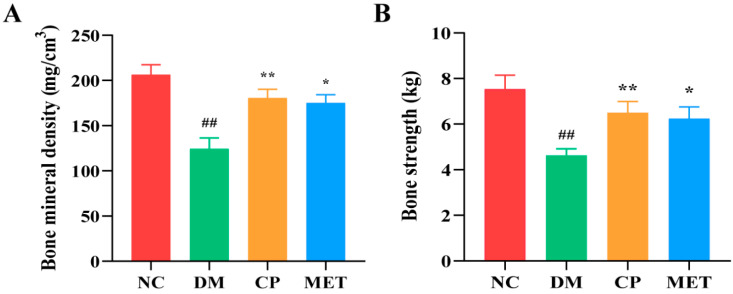
BMD and bone strength of the rats in each group. (**A**) The femur BMD. (**B**) The femur bone strength. The values are presented as mean ± SD. In comparison to the NC group, (##) *p* < 0.01. In comparison to the DM group, (**) *p* < 0.01, (*) *p* < 0.05.

As shown in Figure 2, the BMD and bone strength of the femur in the DM group were remarkedly reduced (*p* < 0.01 vs. NC); However, after administration, femur BMD and bone strength were obviously improved in the CP and MET groups (*p* < 0.05 vs. DM), indicated that skeletal status of diabetic rats was improved after CP administration.

### 2.3. micro-CT Test Results

**Figure 3:**
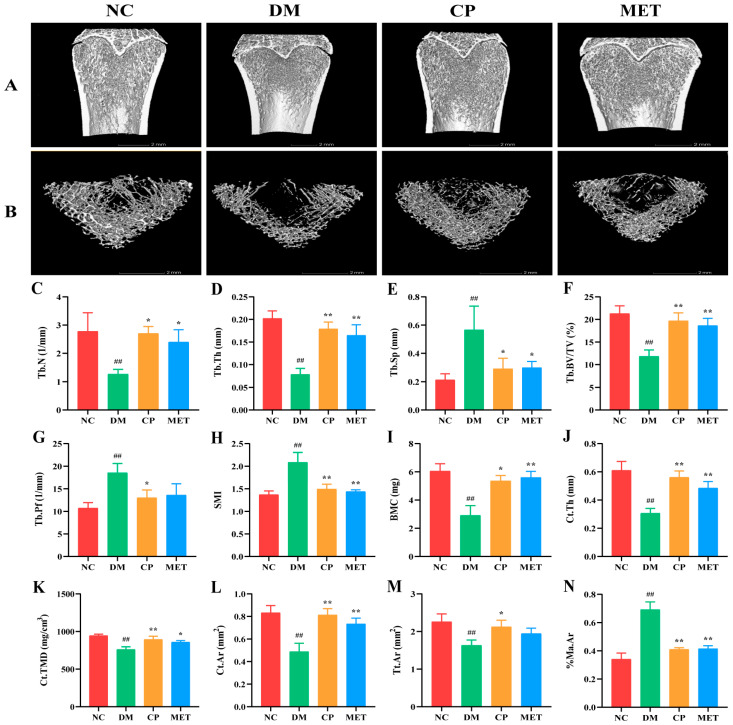
Three-dimensional reconstruction images of femur and bone micro-CT parameters in each group. (**A**) Images of femur. (**B**) Femoral bone cross section. (**C**) Tb.N. (**D**) Tb.Th. (**E**) Tb.Sp. (**F**) Tb.BV/TV. (**G**) Tb.Pf. (**H**) SMI. (**I**) BMC. (**J**) Ct.Th. (**K**) Ct.TMD. (**L**) Ct.Ar. (**M**) Tt.Ar. (**N**) %Ma.Ar. The values are presented as mean ± SD. In comparison to the NC group, (##) *p* < 0.01. In comparison to the DM group, (**) *p* < 0.01, (*) *p* < 0.05.

As indicated in Figure 3, compared with the NC group, the bone trabecular in the DM group was sparse and loose, with a large number of fractures, and trabecular number (Tb.N), trabecular thickness (Tb.Th), relative bone volume (Tb.BV/TV), bone mineral content (BMC), cortical bone thickness (Ct.Th), cortical bone density (Ct.TMD), cortical bone area (Ct.Ar), and total cortical bone area (Tt.Ar) were obviously decreased (*p* < 0.01). After CP intervention, Tb.Th, Tb.BV/TV, Ct.Th, Ct.TMD and Ct.Ar in the CP group were significantly increased (*p* < 0.01 vs. DM); trabecular separation (Tb.Sp), trabecular bone pattern factor (Tb.Pf), structural model index (SMI), and percentage of bone marrow cavity area (%Ma.Ar) were decreased significantly (*p* < 0.05 vs. DM). These results indicated that CP could repair bone structure in diabetic rats.

### 2.4. Chromium Picolinate Repaired Bone Microstructure

**Figure 4:**
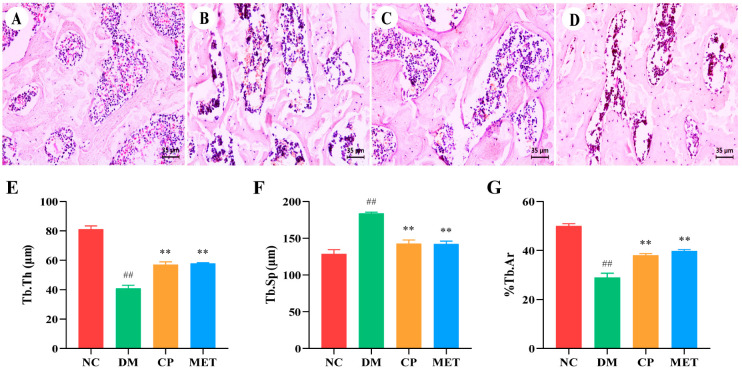
The femoral morphology of the rats in each group. (**A**–**D**) H&E staining of femoral tissues, magnification of 400×; (**E**) Tb.Th of femur; (**F**) Tb.Sp of femur; (**G**) %Tb.Ar of femur. The values are presented as mean ± SD. In comparison to the NC group, (##) *p* < 0.01. In comparison to the DM group, (**) *p* < 0.01.

As indicated in Figure 4, in the NC group, the femoral bone trabecular structure was intact, and the bone trabecular was closely aligned with each other. The femoral bone trabecular structure in DM rats was destroyed; a large number of fractures appeared between the bone trabecular; the percentage of trabecular area (%Tb.Ar) and Tb.Th were obviously reduced (*p* < 0.01 vs. NC); and Tb.Sp was markedly increased (*p* < 0.01 vs. NC). The bone tissues of rats in the CP group were improved, Tb.Th and %Tb.Ar were increased (*p* < 0.05 vs. DM), and Tb.Sp was decreased (*p* < 0.05 vs. DM). Bone microstructure changes in the MET group were similar to the CP group. These results indicated that CP could repair bone microstructure of diabetic rats.

### 2.5. Chromium Picolinate Regulated Bone Turnover

As indicated in Table 1, the levels of OPG and osteocalcin in the DM group were obviously reduced (*p* < 0.01 vs. NC), while the levels of RANKL, TRACP 5b, ALP, and CTX-1 were markedly elevated (*p* < 0.01 vs. NC). After CP intervention, the levels of RANKL, TRACP 5b, ALP, and CTX were markedly decreased (*p* < 0.01 vs. DM). These results indicated that CP could adjust bone turnover in diabetic rats.

### 2.6. Chromium Picolinate Alleviated Oxidative Stress

**Figure 5:**
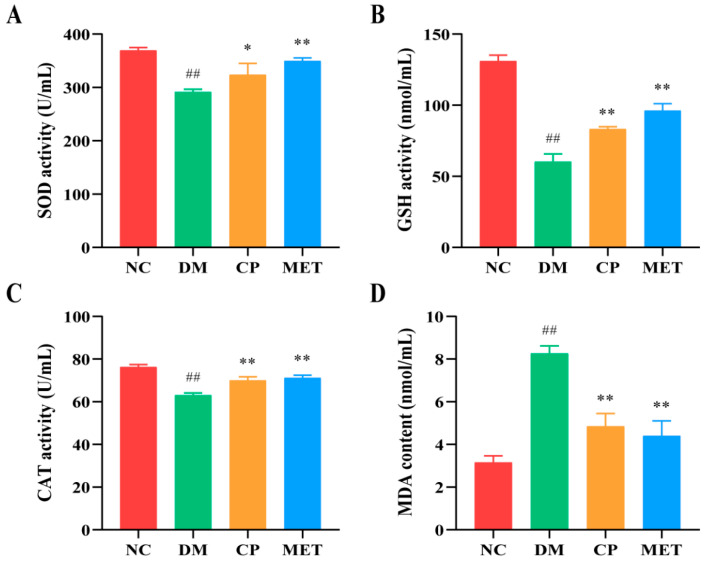
The levels of serum oxidative stress-associated enzymes in each group. (**A**) SOD; (**B**) GSH; (**C**) CAT; (**D**) MDA. The values are presented as mean ± SD. In comparison to the NC group, (##) *p* < 0.01. In comparison to the DM group, (**) *p* < 0.01, (*) *p* < 0.05.

As indicated in Figure 5, the content of antioxidant enzymes (SOD, CAT, and GSH) in the DM group were lower than those of the NC group (*p* < 0.01), and the content of MAD was markedly increased, which indicated that the degree of oxidative stress in diabetic rats was increased. The activity of serum antioxidant enzymes (SOD, CAT, and GSH) was significantly increased after CP treatment (*p* < 0.05 vs. DM), and the content of MAD was obviously reduced after CP treatment (*p* < 0.05 vs. DM), indicated that oxidative stress was improved after CP treatment.

### 2.7. Chromium Picolinate Lowered Serum Inflammatory Cytokines

**Figure 6:**
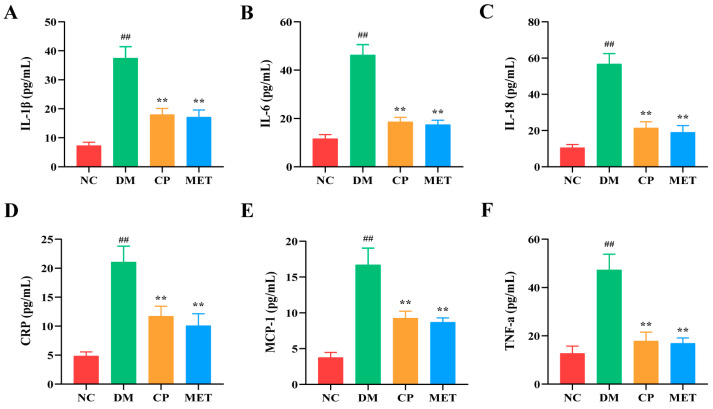
Expression levels of serum inflammatory cytokines in each group. (**A**) IL-1β; (**B**) IL-6; (**C**) IL-18; (**D**) CRP; (**E**) MCP-1; (**F**) TNF-α. The values are presented as mean ± SD. In comparison to the NC group, (##) *p* < 0.01. In comparison to the DM group, (**) *p* < 0.01.

As indicated in Figure 6, the levels of serum interleukin-1β(1IL-1β), interleukin-6 (IL-6), interleukin-18 (IL-18), C-reactive protein (CRP), and monocyte chemoattractant protein-1 (MCP-1) in DM group were markedly increased (*p* < 0.05 vs. NC). After CP intervention, the levels of those serum inflammatory cytokines were decreased (*p* < 0.05 vs. DM). These results indicated that CP could reduce the content of serum inflammatory cytokines in diabetic rats.

### 2.8. Chromium Picolinate Inhibited Bone Marrow Adipocyte Differentiation

**Figure 7:**
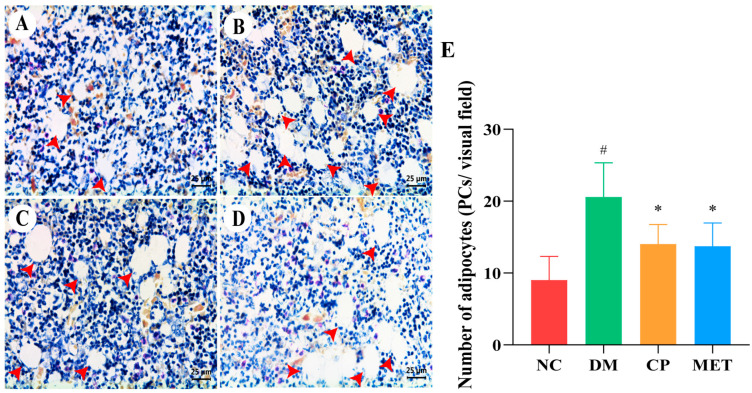
H&E staining, magnification of 400×. (**A**) Tibia of NC group. (**B**) Tibia of DM group. (**C**) Tibia of CP group. (**D**) Tibia of MET group. (**E**) Adipocytes numbers in tibial tissue of rats in each group. The red arrow points to adipocytes. The values are presented as mean ± SD. In comparison to the NC group, (#) *p* < 0.05. In comparison to the DM group, (*) *p* < 0.05.

As indicated in Figure 7, in the NC group, there was a large distribution of bone marrow in the tibia, with a small number of adipocytes. The number of tibial adipocytes in the DM group was markedly increased (*p* < 0.05 vs. NC). The number of tibial bone marrow adipocytes was obviously decreased in CP and MET groups (*p* < 0.05 vs. DM). These results indicated that CP could inhibit adipocyte differentiation in bone marrow of diabetic rats.

### 2.9. Chromium Picolinate Inhibited Osteoclastogenesis 

**Figure 8:**
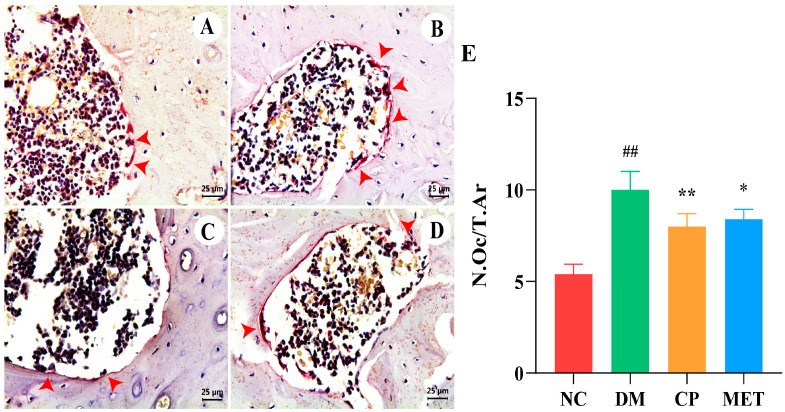
TRAP staining, magnification of 400×. (**A**) Femur bone of NC group. (**B**) Femur bone of DM group. (**C**) Femur bone of CP group. (**D**) Femur bone of MET group. (**E**) Osteoclast numbers in each group. The red arrows point to osteoclasts. The values are presented as mean ± SD. In comparison to the NC group, (##) *p* < 0.01. In comparison to the DM group, (**) *p* < 0.01, (*) *p* < 0.05.

As indicated in Figure 8, TRAP staining showed that osteoclasts in the rat femur were purplish red and distributed along the edge of trabecular bone. The number of femoral osteoclasts in the DM group was markedly increased (*p* < 0.01 vs. NC). In the CP group and MET group, the number of osteoclasts was decreased significantly (*p* < 0.05 vs. DM), indicated that CP could inhibit osteoclastogenesis in diabetic rats.

### 2.10. Chromium Picolinate Promoted Osteoblastogenesis 

**Figure 9:**
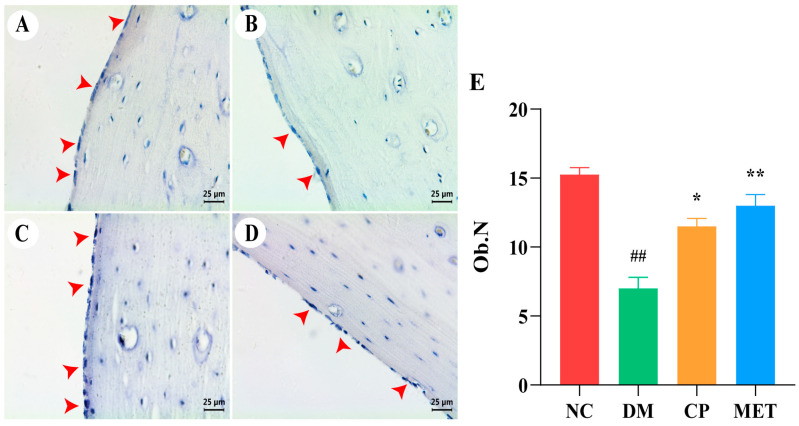
ALP staining, magnification of 400×. (**A**) Femur bone of NC group. (**B**) Femur bone of DM group. (**C**) Femur bone of CP group. (**D**) Femur bone of MET group. (**E**) Osteoblasts numbers in each group. The red arrows point to osteoblasts. The values are presented as mean ± SD. In comparison to the NC group, (##) *p* < 0.01. In comparison to the DM group, (**) *p* < 0.01, (*) *p* < 0.05.

As shown in Figure 9, ALP staining showed that femoral osteoblasts in DM rats were mainly distributed around the trabecular bone and were sparsely arranged, and the number of osteoblasts was obviously reduced in DM rats (*p* < 0.01 vs. NC). After CP or MET administration, the number of femoral osteoblasts in CP and MET groups was significantly increased (*p* < 0.01 vs. DM).

### 2.11. Chromium Picolinate Promoted Bone Collagen Fibers Formation 

**Figure 10:**
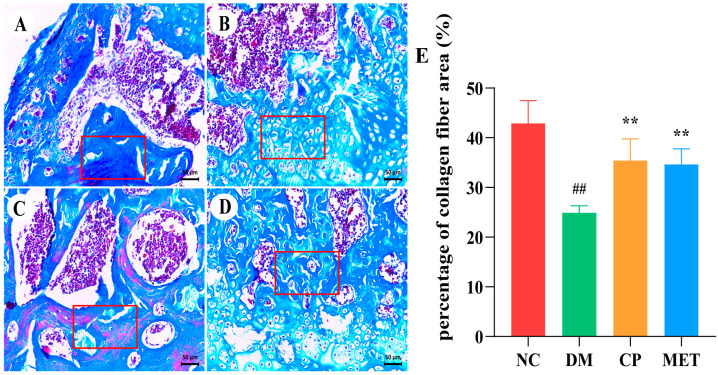
Masson staining, magnification of 200×. (**A**) Femur bone of NC group. (**B**) Femur bone of DM group. (**C**) Femur bone of CP group. (**D**) Femur bone of MET group. (**E**) Percentage of collagen fiber area (%) in each group. The red box shows collagen fibers. The values are presented as mean ± SD. In comparison to the NC group, (##) *p* < 0.01. In comparison to the DM group, (**) *p* < 0.01.

Masson trichrome staining was performed on femoral bone tissues to observe the bone collagen fibers, and the results are shown in Figure 10. Collagen fibers were widely distributed in the femoral bone tissue of the NC group, and the proportion of collagen fiber area was high, whereas the percentage of bone collagen fiber area in DM rats was markedly reduced (*p* < 0.01 vs. NC). The percentage of collagen fiber area in the CP and MET groups were obviously increased (*p* < 0.05 vs. DM), indicating that CP could promote the formation of bone collagen in diabetic rats.

### 2.12. Effect of Chromium Picolinate on Expression of OPG, RUNX 2 as Well as RANKL in Bone Tissue

**Figure 11:**
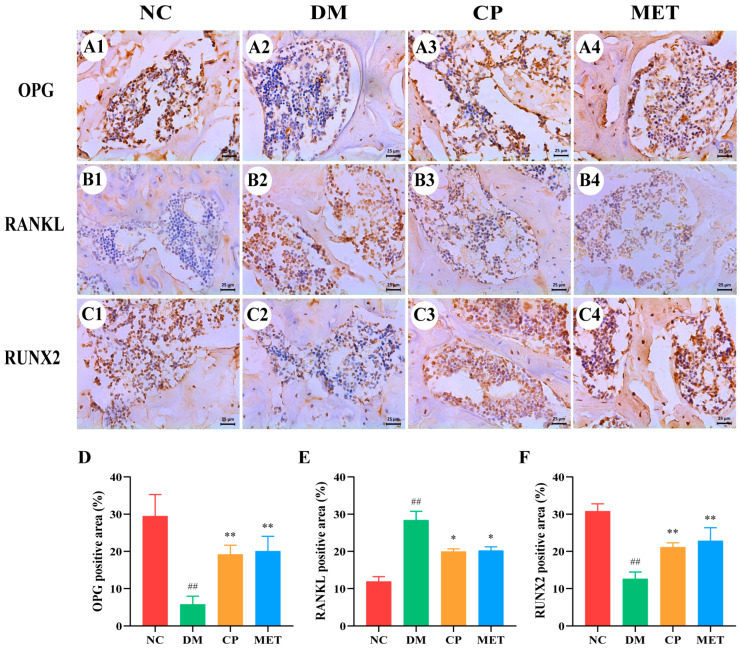
Expression of OPG, RANKL as well as RUNX 2 proteins in bone tissue of rats in each group, with IHC staining and magnification of 400×. (**A1**–**A4**) OPG; (**B1**–**B4**) RANKL; (**C1**–**C4**) RUNX 2; (**D**) OPG positive staining area percentage; (**E**) RANKL positive staining area percentage; (**F**) RUNX 2 positive staining area percentage. The values are presented as mean ± SD. In comparison to the NC group, (##) *p* < 0.01. In comparison to the DM group, (**) *p* < 0.01, (*) *p* < 0.05.

As indicated in Figure 11, the expression of OPG protein and RUNX 2 protein were obviously lower in the DM group than those in the NC group (*p* < 0.01), but they were markedly increased after 8 weeks of CP or MET interventions (*p* < 0.05 vs. DM); RANKL protein was at a high level in the DM group (*p* < 0. 01 vs. NC), and it was markedly lowered in the CP and MET groups (*p* < 0.05 vs. DM). These results indicated CP could regulate the expression of bone metabolism-related proteins.

## 3. Discussion

The degree of cell damage caused by STZ in animals of the same genus depends on the dosage of STZ. Intraperitoneal or intravenous injection of STZ 40–90 mg/kg in rats can directly damage pancreatic islet β cells, so it has been widely used by researchers for diabetic animal model construction [22]. STZ can specifically damage pancreatic beta cells in a short time and reduce insulin production in the body [23]. Therefore, here we gave rats a one-time intraperitoneal injection of STZ (45 mg/kg). After 72 h, the blood glucose levels of rats were increased significantly, 39 of the 42 rats injected with STZ had blood glucose above 11.1 mmol/L, and the diabetes model construction success rate reached 93%, which was consistent with our previous studies [24,25]. After 8 weeks, the bone strength, bone mass, and bone mineral density of these rats were decreased, indicating that the diabetic osteoporosis model was successfully established. At the same time, compared with diabetic rats, water and food intake levels were definitely lower in the control group, and the weight of the control group was definitely higher than that of diabetes rats. Similar changes were found in all animal experimental studies on diabetes.

Studies have shown that supplementing CP could improve insulin sensitivity in obese people and diabetic patients, improve glucose disposal rates, and achieve hypoglycemic effects [20,26]. Thus, CP has been widely used in the fields of hypoglycemic functional foods and pharmaceuticals. In addition, CP has been confirmed to have a positive effect on diabetic complications, such as protecting diabetic atherosclerosis by inhibiting the expression of Thrombospondin-1, improving diabetic nephropathy and coronary heart disease through antioxidant and anti-inflammation effects [21,27,28,29]. Here we administered CP to diabetic rats for 8 weeks. The results showed that CP could improve bone metabolism disorders, bone loss, and bone tissue microstructural destruction in diabetic rats, thereby preventing diabetic osteoporosis. At the same time, in this study, metformin was used as the positive control; here we observed the protective effect of metformin on diabetic osteoporosis, which was consistent with other reports [30,31,32], indicated that our research methods were reliable.

The results of micro-CT were consistent with those of H&E staining of femur tissues; bone trabeculae were sparsely arranged, and fractures were more frequent in the DM group. After 8 weeks of CP intervention, the bone microstructure was improved. At the same time, the results of bone histomorphology measurement were consistent with the micro-CT results, all these results showed that CP could improve diabetes caused bone trabecular structure damage and bone loss.

The activity of bone formation and bone resorption can be evaluated by observing the expression of bone turnover markers, which were also used in osteoporosis diagnosis and treatment. TRACP 5b is released when osteoclasts absorb bone and digest bone proteins (such as CTX 1); osteoblasts secrete osteocalcin and ALP when they work, and this process is regulated by RUNX 2 [33,34]. We found that bone formation biochemical indices such as osteocalcin and ALP were significantly increased in diabetic rats after CP intervention, and bone resorption biochemical indices such as TRACP 5b and CTX 1 were significantly decreased. This indicates that CP could regulate bone turnover.

When osteoporosis occurred, the number of bone marrow mesenchymal stem cells differentiating into adipocytes increases and osteoblast generation decreases [35,36,37,38]; studies have shown that activation of bone morphogenetic protein 2 could inhibit adipogenesis [39,40]. In this study, pathological staining of bone tissue showed that the number of adipocytes in the tibial bone marrow of the DM group was markedly increased, and the number of osteoblasts was markedly decreased. The differentiation of adipocytes was decreased, and the differentiation of osteoblasts was increased in CP rats. Studies have shown that metformin, melatonin, or exercise intervention could also reduce the number of bone marrow adipocytes, thus increasing the BMD [41,42,43]. This was consistent with our research.

Meanwhile, collagen tissue can increase BMD and osteoblast activity, which has a positive effect on bone strength [44,45,46]. Seo, J.Y. et al. have shown that 7α,25-dihydroxycholesterol reduced the level of type II collagen in chondrocytes, thus accelerating proteoglycan loss and ultimately inducing osteoarthritis [47]. Huang W et al. found that the bone defect disappeared in a rabbit iliac bone defect model after 4 weeks’ treatment with catechol-conjugated chitosan multi-functional hydrogel, and massive new bone tissue in the iliac bone could be observed in Masson trichrome [48]. In this study, Masson trichrome of bone tissue showed that CP intervention could significantly increase the bone collagen fiber area of rats and improve the dissolution, fracture, and loose arrangement of collagen fibers.

Sustained hyperglycemia will cause the overproduction of ROS in the body; destroy the homeostasis of antioxidant enzymes and oxidative enzymes; cause oxidative stress, thus activating the differentiation of osteoclast precursors; enhance osteoclast bone resorption; and accelerate bone loss [49,50,51]. High blood glucose concentrations will stimulate the release of inflammatory mediators, while oxidative stress will also upregulate inflammatory cytokines, and inflammation, in turn, will promote the generation of ROS [52,53]. In this study, the levels of serum SOD, CAT, and GSH in the DM group were remarkably reduced, while MAD content and inflammatory cytokine levels were significantly increased. After administration of CP, the antioxidant enzyme activity in the serum of diabetic rats markedly improved, and MAD content and inflammatory cytokine levels were decreased significantly. The results showed that CP could prevent bone loss caused by diabetes by inhibiting oxidase activity and anti-inflammation. At the same time, studies have confirmed that Liuwei Dihuang Pill or puerarin and other drugs could improve bone loss by reducing the level of inflammatory cytokines in diabetic rats [54,55], which were consistent with our study. Bo, S. et al. showed that taking resveratrol in diabetic patients could increase the level of antioxidant markers and improve the decline of bone mineral density [56]. This is consistent with our study, suggesting that antioxidant, and anti-inflammation are some of the mechanisms of CP anti-diabetic osteoporosis.

The OPG/RANKL/RANK signaling pathway adjusts bone formation and bone resorption [57]. RANKL activates osteoclast differentiation and binds to the receptor RANK to promote bone resorption [58]. OPG binds to the binding site of RANKL, inhibits RANK binding and activation, and inhibits osteoclast differentiation [59]. The ratio of OPG/RANKL reflects the trend of bone transformation in the body. When the ratio decreases, it indicates that osteoclast proliferation is active and bone resorption is promoted [60]. We examined OPG, RANKL, and RUNX 2 proteins, and found that after CP intervention, the expression of OPG and RUNX 2 proteins were increased, while the expression of RANKL protein was decreased, OPG/RANKL ratio was increased. These results indicated that CP could alleviate DOP by regulating the OPG/RANK/RANKL signaling pathway.

## 4. Materials and Methods

### 4.1. Chemicals

STZ was bought from Beijing Coolaibo Technology Company Limited (Beijing, China). Chromium picolinate was bought from Sinopharm Chemical Reagent Company (Shanghai, China). Rabbit anit-OPG, anit-RANKL, and anit-RUNX 2 were bought from Beijing Bioss Antibodies Company Limited (Beijing, China). Goat-Rabbit IgG was bought from Beijing TDY Biotechnology Company Limited (Beijing, China).

### 4.2. Animals

Male SD rats aged 56 days (*n* = 52) were purchased from Cheng Du Dashuo Experimental Animal Company (Chengdu, China). All animals were housed one week before the experiment under standardized conditions. Animals were provided adequate food and water. Animal experiments were approved by the local animal ethics committee (approval No. 2022-14).

### 4.3. Animal Grouping

After one week of adaptive feeding, 52 healthy male SD rats were randomly divided into two groups, the NC group (*n* = 10) rats were injected with citrate buffer, and other rats (*n* = 42) were injected with STZ 45 mg·kg^−1^ peritoneally (the dosage of STZ was selected based on our previous research). After 72h, the rats (*n* = 39) with blood glucose above 11.1 mmol·L^−1^ were re-divided into 3 groups. For 8 weeks, the NC group and diabetic model group (DM group, *n* = 13) were intragastrically given distilled water, the chromium picolinate group (CP group, *n* = 13) was intragastrically given 5 mg·kg^−1^·d^−1^ CP, and the metformin group (MET group, *n* = 13) was intragastrically given 200 mg·kg^−1^·d^−1^ metformin.

The blood glucose, body weight, water intake, and food intake were recorded on the last day of each week for each group of rats. After 8 weeks, the rats were killed by cervical dislocation, and blood was collected from orbital venous plexus. The blood was centrifuged and stored in the refrigerator at −80 °C. Two femur and tibia tissues of each rat were collected, and one of them was fixed in 4% paraformaldehyde, and the other was frozen in a −80 °C refrigerator.

### 4.4. BMD and Bone Strength Measurement 

The BMD of the femur in each rat was determined using BMD and Body Composition Analysis System for Lab Animals (KUBTEC Technologies INC., Stratford, CT, USA). The bone strength of the femur of each rat was determined using a Small Animal Bone Strength Tester (Anhui Zhenghua Biologic Apparatus Facilities, Huaibei, China).

### 4.5. Bone Micro-CT Detection

The connective tissue on the surface of the rat femur was removed, and then X-ray scanning was performed using a micro-CT instrument (Bruker (Beijing) Scientific Technology Co., Ltd., Beijing, China). The scanning voltage was 70 kV, the scanning current was 114 μA, and the scanning time was 29.7 min [61]. After scanning, the bone tissue region 1.0 mm distal to the growth plate was selected for 3D reconstruction. Bone structure was analyzed according to all measurement parameters listed in Table 2.

### 4.6. Histopathological Analysis of Femur Sections

Rat femur tissues were fixed and decalcified for 28 days. Paraffin sections (5 µm thick) were prepared with a tissue microtome (Leica, Wetzlar, Germany) and stained with TRAP, ALP, hematoxylin-eosin, and Masson trichromatic. Stained slides were viewed under a microscope and photographed from five different viewing angles using a Leica Photography system. The software program Image J 1.52 was used to measure Tb.Th, Tb.Sp, the number of osteoclasts, osteoblasts, and the collagen fiber area in bone tissues in each visual field. According to the calculation formula of bone morphometry, trabecular %Tb.Ar were calculated [62].

### 4.7. Oxidative Stress, Bone Turnover Markers and Inflammatory Cytokines Detection

According to the instructions of the kit (Beijing Solarbio Science & Technology Co., Ltd., Beijing, China), the microplate reader (Nanjing Detie Biotechnology Co., Ltd., Nanjing, China) and ultra-violet and visible spectrophotometer (Shimadzu Enterprise Management (China) Co., Ltd., Shanghai, China) were used to detect oxidative stress (SOD, MDA, CAT, and GSH), bone turnover markers (OPG, CTX-1, TRACP 5b, ALP, RANKL, osteocalcin, and RUNX 2) and inflammatory cytokines (IL-8, TNF-α, IL-6, IL-1β, MCP-1 and CRP) levels in serum of rats.

### 4.8. Bone Marrow Adipocytes in the Tibia

After rat tibia tissues were fixed and decalcified for 28 days, paraffin sections (5 µm thick) were prepared with a tissue microtome and stained with hematoxylin and eosin. The number of adipocytes in tibia bone tissues were counted under a microscope from ten different viewing angles using the Leica Photography system.

### 4.9. Immunohistochemical (IHC)

The paraffin sections of femur were dewaxed with xylene and dehydrated, soaked in 1%TritonX-100 for 30 min, soaked in CBS at 95 °C for 20 min, then cooled naturally, soaked in 0.3% hydrogen peroxide solution for 30min, and washed with PBS. Non-specific antigens were blocked with 3%BSA solution. The femur sections were then washed with PBS; OPG, RANKL and RUNX 2 primary antibody (Beijing Bioss Biotechnology Co., Ltd., Beijing, China) were added for 90 min and incubated at 37 °C. After PBS washing, drops of secondary antibody (TDY biotech, Beijing, China) were added and incubated for 2 h. After washing with PBS, DAB was added, the nucleus was stained with hematoxylin, and the slides were sealed with neutral gum. In each slide, we selected ten different fields of view and calculated the proportion of positive staining areas with Image J 1.52.

### 4.10. Data Analysis

SPSS (version 22.0) was used to analyze the data. Univariate analysis of variance (ANOVA) and Tukey’s test were used to analyze the differences among the groups. *p* < 0.05 indicated statistically significant.

## 5. Conclusions

In sum, this study found that CP could prevent DOP, increase BMD, improve bone tissue structure, increase the number of bone collagen fibers and osteoblasts, and reduce bone marrow adipocyte and osteoclast production. In addition, CP could inhibit oxidative stress and inflammation and upregulate the expression of OPG and RUNX 2 proteins, as well as balance the level of bone turnover markers. These effects are conducive to maintaining normal bone metabolic balance and alleviating DOP. Thus, CP could be used as an effective ingredient for DOP treatment.

## Figures and Tables

**Table 1:** Bone turnover markers in serum of different groups.

Parameter	NC	DM	CP	MET
OPG (ng mL^−1^)	8.93 ± 1.07	3.37 ± 0.69 ^##^	7.04 ± 1.26 **	7.33 ± 0.97 **
RANKL (ng mL^−1^)	4.01 ± 0.66	11.37 ± 1.97 ^##^	5.17 ± 0.74 **	5.49 ± 0.67 **
OPG/RANKL ratio	2.44 ± 0.47	0.31 ± 0.11 ^##^	1.51 ± 0.39 **	1.49 ± 0.37 **
RUNX 2 (ng mL^−1^) TRACP 5b (U dL^−1^)	9.57 ± 1.89	3.41 ± 0.79	8.46 ± 1.27 **	7.96 ± 1.83 **
	2.29 ± 0.63	7.32 ± 1.43 ^##^	3.37 ± 1.21 **	4.01 ± 1.34 *
ALP (U dL^−1^) CTX-1 (ng mL^−1^) Osteocalcin (ng mL^−1^)	59.64 ± 7.97	137.35 ± 14.31 ^##^	77.12 ± 8.45 **	84.36 ± 10.67 **
	35.47 ± 5.21	101.47 ± 11.69 ^##^	66.47 ± 8.74 **	61.77 ± 9.14 **
	26.78 ± 4.12	8.45 ± 2.17 ^##^	19.67 ± 3.64 **	21.76 ± 3.99 **

The values are presented as mean ± SD. In comparison to the NC group, (^##^) *p* < 0.01. In comparison to the DM group, (**) *p* < 0.01, (*) *p* < 0.05.

**Table 2:** Bone tissue measurement parameters detected by micro-CT.

Type of Analysis	Parameter	Unit
Trabecular bone—3D imaging	Trabecular number (Tb.N)	1/mm
	Trabecular thickness (Tb.Th)	mm
	Trabecular separation (Tb.Sp)	mm
	Relative bone volume (Tb.BV/TV)	%
	Trabecular bone pattern factor (Tb.Pf)	1/mm
	Structural model index (SMI)	
	Bone mineral content (BMC)	mg
Cortical bone—3D imaging	Cortical bone thickness (Ct.Th)	mm
	Cortical bone density (Ct.TMD)	mg/cm^3^
	Cortical bone area (Ct.Ar)	mm^2^
	Total cortical bone area (Tt.Ar)	mm^2^
	Percentage of bone marrow cavity area (%Ma.Ar)	%

## Data Availability

The data presented in this study are available in article.
